# 49th Annual Meeting of the Indian Society of Human Genetics and International Conclave on Neurogenetics (ISHG-2025): India’s march towards low-cost rare disease therapeutics

**DOI:** 10.1016/j.lansea.2025.100632

**Published:** 2025-07-05

**Authors:** Mathivanan Jothi, B.K. Thelma, Monojit Debnath

**Affiliations:** a Department of Human Genetics, National Institute of Mental Health and Neurosciences, Bengaluru, India; b Department of Genetics, University of Delhi South Campus, New Delhi, India

The 49th Annual Meeting of the Indian Society of Human Genetics and the International Conclave on Neurogenetics (ISHG-2025) was organised by the Department of Human Genetics at the National Institute of Mental Health and Neurosciences (NIMHANS), Bengaluru, India, during Jan 20–22, 2025. This conference brought together over 650 delegates from multiple countries to discuss advancements in human genetics, with a special focus on neurogenetics, epigenetics, discovery genomics, precision medicine, gene therapy, and animal modelling of genetic diseases.

The inaugural address underscored the significant role of Indian genetic researchers in driving innovation and developing cost-effective products, including small molecules for the treatment of rare diseases. It also clearly outlined the Government of India’s roadmap and steadfast support for rare disorder therapeutics. NITI Aayog is working closely with a team of geneticists and clinicians to develop therapies for rare genetic diseases, such as small molecule drugs, gene therapy, and enzyme replacement therapy, at affordable costs. Thirteen rare diseases, including Gaucher’s disease, Wilson’s disease, tyrosinemia type 1, phenylketonuria, cystic fibrosis, hyperammonaemia, and Lennox-Gastaut syndrome, have been prioritised for small molecule development, while spinal muscular atrophy, Duchenne muscular dystrophy, achondroplasia, mucopolysaccharidosis, Pompe’s disease, and Neimann-Pick disease have been prioritised for gene or enzyme replacement therapy. The team has been working on nine drugs; to date, four drugs have been developed in India and are available at significantly reduced prices. These include nitisinone for tyrosinemia type 1, eliglustat for Gaucher’s disease, trientine for Wilson’s disease, and cannabidiol (oral solution) for Lennox-Gastaut syndrome. Efforts are also underway to establish a Cell and Gene Therapy Mission, involving both the scientific community and industry partners.

Since the launch of the National Sickle Cell Anaemia Elimination Mission by the Prime Minister of India on July 1, 2023, approximately 50 million people in India have been screened for this condition. Hydroxyurea syrup, manufactured in India, will be integrated into the elimination mission, with each unit costing INR 1500 (US$ 17.46 as of June 17, 2025). Multi-centre efforts are needed to address the challenges of rare genetic disorders and to make gene therapies accessible and affordable globally. The need to understand the genetic basis of conditions with significant public health implications, such as alcohol-induced disorders, was also highlighted.

A panel discussion featuring seven leading Indian geneticists provided valuable guidance to the scientific community, industry stakeholders, and policymakers. Key recommendations included promoting research on gene therapies for rare genetic disorders beyond immunodeficiency and haematological diseases, and increasing pharmaceutical industry involvement in large-scale vector production to accelerate gene therapy development. Given India’s large population, genetic heterogeneity, and practices such as endogamy, it was suggested that the Indian Council of Medical Research expand the range of diseases eligible for funding to include both rare and complex conditions. The integration of ‘phenomics’ (comprehensive phenotype profiling) into the spectrum of OMICS-based research, especially utilising artificial intelligence, was also proposed for more precise disease association and causality studies.

Attention was drawn to the need for a national biobank, tailored to address emerging challenges posed by early-onset lifestyle diseases, which would require public-private partnership for establishment. The importance of developing regulatory guidelines for pharmacogenomics and next-generation sequencing-based genetic testing was emphasised, recommending collaboration between the Indian Society of Human Genetics and the Society for Indian Academy of Medical Genetics. Implementing a non-discrimination act for genomics, essential for secure genetic data sharing, was also highlighted. The Genome India Project’s sequencing of 10,000 genomes may provide leads for disease burden, and personalised medicine.

The theme of the conference was focused on key advances in genetics and genomics. Topics addressed included the diversity of mutations and their links to human diseases, the transformative impact of genetics in medicine, the effectiveness of emerging technologies for unsolved neurological disorders, the influence of human genetic variations on disease susceptibility and drug reactions, and the crucial role of genomic tools in precision medicine, as well as the importance of equitable access to genomics for global health.

In summary, ISHG-2025 provided a platform for sharing ground-breaking research in human genomics, offering meaningful insights into innovations in genomic tools and therapies, understanding the genetic landscape of rare and complex diseases, exploring cost-effective management options, and prospects in genomic medicine.

## Contributors

MJ, BKT, and MD: Conceptualisation of the idea and drafting the manuscript.

## Declaration of interests

We declare no competing interests.

